# Aging Attitude and Post-retirement Re-employment Among Retired and Unretired Hong Kong Older Adults

**DOI:** 10.1093/geroni/igaf122.3882

**Published:** 2025-12-31

**Authors:** Yue Hu, Helene Fung, Chunyan Mai

**Affiliations:** The Chinese University of Hong Kong, Hong Kong, China (People’s Republic); The Chinese University of Hong Kong, Hong Kong, China (People’s Republic); The Chinese University of Hong Kong, Hong Kong, China (People’s Republic)

## Abstract

Literature about views on aging and preparation for late life indicates the pivotal role of attitude towards aging in planning for post-retirement reemployment. However, it still remains unclear whether negative age stereotypes impede post-retirement re-employment among retired and unretired older adults. Using a cross-sectional design, 317 Hong Kong older adults (50 – 80 yrs, mean age 60.99 ± 6.17, 54% females, 113 unretired) were recruited by using a convenience sampling method. We found that unretired older adults’ negative attitudes toward older population in personality and mental health predicted a lower likelihood of post-retirement employment planning at a marginally significant level (p = .07), even after controlling socioeconomic status. Retired older adults’ negative attitudes toward older adult population in their societal participation were significantly associated with a lower likelihood of actual post-retirement employment (p = .03 < .05). Meanwhile, negative attitudes toward the older adult population in their physical aspects were significantly associated with a higher likelihood of actual post-retirement employment (p = 0.002 < .01). Findings suggest that societal expectations about older adults’ vulnerability in personality, mental health and social participation may hamper their productivity in late life. Future studies are warranted to further examine how self-perceptions about aging in different domains shape post-retirement employment planning in a larger sample.

